# Three-dimensional branch segmentation and phenotype extraction of maize tassel based on deep learning

**DOI:** 10.1186/s13007-023-01051-9

**Published:** 2023-08-01

**Authors:** Wenqi Zhang, Sheng Wu, Weiliang Wen, Xianju Lu, Chuanyu Wang, Wenbo Gou, Yuankun Li, Xinyu Guo, Chunjiang Zhao

**Affiliations:** 1 Information Technology Research Center, Beijing Academy of Agriculture and Forestry Sciences, Beijing, 100097 China; 2 Beijing Key Lab of Digital Plant, National Engineering Research Center for Information Technology in Agriculture, Beijing, 100097 China; 3 College of Information Technology, Shanghai Ocean University, Shanghai, 201306 China

**Keywords:** Maize tassel, Point cloud, Deep learning, Organ segmentation, Tassel phenotype, DUS testing

## Abstract

**Background:**

The morphological structure phenotype of maize tassel plays an important role in plant growth, reproduction, and yield formation. It is an important step in the distinctness, uniformity, and stability (DUS) testing to obtain maize tassel phenotype traits. Plant organ segmentation can be achieved with high-precision and automated acquisition of maize tassel phenotype traits because of the advances in the point cloud deep learning method. However, this method requires a large number of data sets and is not robust to automatic segmentation of highly adherent organ components; thus, it should be combined with point cloud processing technology.

**Results:**

An innovative method of incomplete annotation of point cloud data was proposed for easy development of the dataset of maize tassels,and an automatic maize tassel phenotype analysis system: MaizeTasselSeg was developed. The tip feature of point cloud is trained and learned based on PointNet +  + network, and the tip point cloud of tassel branch was automatically segmented. Complete branch segmentation was realized based on the shortest path algorithm. The Intersection over Union (IoU), precision, and recall of the segmentation results were 96.29, 96.36, and 93.01, respectively. Six phenotypic traits related to morphological structure (branch count, branch length, branch angle, branch curvature, tassel volume, and dispersion) were automatically extracted from the segmentation point cloud. The squared correlation coefficients (R^2^) for branch length, branch angle, and branch count were 0.9897, 0.9317, and 0.9587, respectively. The root mean squared error (RMSE) for branch length, branch angle, and branch count were 0.529 cm, 4.516, and 0.875, respectively.

**Conclusion:**

The proposed method provides an efficient scheme for high-throughput organ segmentation of maize tassels and can be used for the automatic extraction of phenotypic traits of maize tassel. In addition, the incomplete annotation approach provides a new idea for morphology-based plant segmentation.

## Introduction

Plant phenotype helps in understanding plant environmental interactions and translating their applications in crop management, biostimulants, microbial communities, etc. [1–3]. Traditional phenotypic measurements are manually conducted at the single plant level, which is inefficient and has low precision, thus limiting the analysis of the genetics of quantitative traits, especially those associated with yield and stress tolerance [4]. Therefore, high-throughput and automated phenotypic measurement techniques should be developed. Maize is grown extensively around the world as an important food and feed crop, and is one of the three major food crops in the world, along with wheat and rice [5]. An important feature of maize is the structure of the male tassels. Maize tassels produce pollen necessary for maize reproduction, and factors such as the number and length of tassel branches are related to factors affecting grain yield [6]. Studies have shown that the yield of plants with removed male tassels is 50.6% higher than that of intact plants, and that smaller male tassels usually release more energy for grain production and reduce light shading, making a good male structure one of the components of an ideal plant type [7]. In addition, regional trials are an important link in breeding and new variety promotion, and manual investigation of the distinctness, uniformity, and stability (DUS) testing traits is a time-consuming and laborious work [8]. The DUS testing traits related to maize tassels include spindle length, number of branches, tassel weight, tassel density, branch length and branch angle, among which, tassel spindle length and branch number are two important traits. Therefore, the tassel structure should be comprehensively evaluated and understood.

In the field planting environment,maize tassels can be recognized, counted and located based on image depth learning technology [9–11], and the point cloud data of maize tassels is incomplete using lidar [12]. Therefore, in order to obtain the three-dimensional morphological phenotypic traits of male tassel, it is necessary to manually sample from the field, and be rapidly obtained via high-throughput data collection platforms. In the indoor environment, Gage obtained maize tassel phenotypic traits, such as the number of branches, tassel length, etc.by the single image technology [13]. These image-based methods have greatly enhanced the development of phenotypic studies of maize tassels. However, two-dimensional-based methods cannot accurately acquire phenotypic traits in three dimensions. As a result, 3D reconstruction methods, including depth camera-based methods [14, 15], LIDAR-based methods [16, 17], and multi-view image reconstruction-based methods [18–20] have been developed to avoid data loss in dimensionality. The 3D data acquisition methods avoid plant self-obscuration that may be encountered in 2D images and provide a basis for complex plant phenotype extraction. The 3D processing software is the traditional method widely used to obtain plant phenotypic traits (leaf area, length, width, inclination, etc.) from 3D data via manual operations, such as organ segmentation on plant 3D data. However, this method is time-consuming and hinders the efficiency of high-throughput phenotype acquisition. Therefore, a method that can improve the efficiency of phenotype extraction is necessary [21]. The DBSCAN(density-based spatial clustering of applications with noise) algorithm is applied to automatic branch segmentation of maize tassel point cloud, but it was difficult to achieve branching and segmentation for compact tassels, so more robust algorithms need to be studied [22]. The DFSP(distance field-based segmentation pipeline) algorithm was proposed for automated segmentation of corn plant stem and leaf point clouds in different directional structures [23].

Automated segmentation techniques based on 3D deep learning can be used to efficiently acquire phenotypic data. The 3D deep learning networks can be divided into three broad categories: 3D voxel grid-based frameworks [24], convolution-based methods [25–27], and the framework for direct input point clouds [28]. PointNet extracts features in a way that a global feature is extracted for all point cloud data without considering the direct relationship between local point clouds [28]. PointNet +  + can extract local features at different scales of point clouds and obtain deep features through a multilayer network structure [29]. Meanwhile, several researchers have used deep learning of 3D point clouds in plant organ segmentation.A novel pattern-based deep neural network Pattern-Net was designed for the segmentation of wheat point clouds [30]. DeepSeg3DMaize was developed to extract six phenotypic traits of maize based on PointNet [31]. A dual-functional deep learning neural network PlantNet was proposed for semantic segmentation and instance segmentation of two dicotyledons and one monocotyledon from point clouds [32].

However, there are a few publicly available datasets for training, which limits the development of deep learning techniques in the plant domain [33]. ROSE-X dataset was developed, which consisted of only 11 annotated three-dimensional models of plants of the genus Rosaceae, and thus is not applicable to other morphological plant species [34]. Therefore, a quick method of annotating plant point cloud data to build data integration should be developed. In this study, an innovative incomplete annotation method was proposed for the rapid construction of point cloud annotation dataset of maize tassels. MaizeTasselSeg, an automated maize tassel point cloud processing system using PointNet +  + as a deep learning model framework to segment the tips of maize tassel branches, was then developed. The shortest path growth algorithm was also proposed for branch segmentation of maize tassels to achieve automated organ-level phenotypic trait extraction of maize tassels. This study provides an automated and efficient solution for the 3D phenotypic analysis of maize tassels.

## Materials and methods

### Overview

**Fig. 1:**
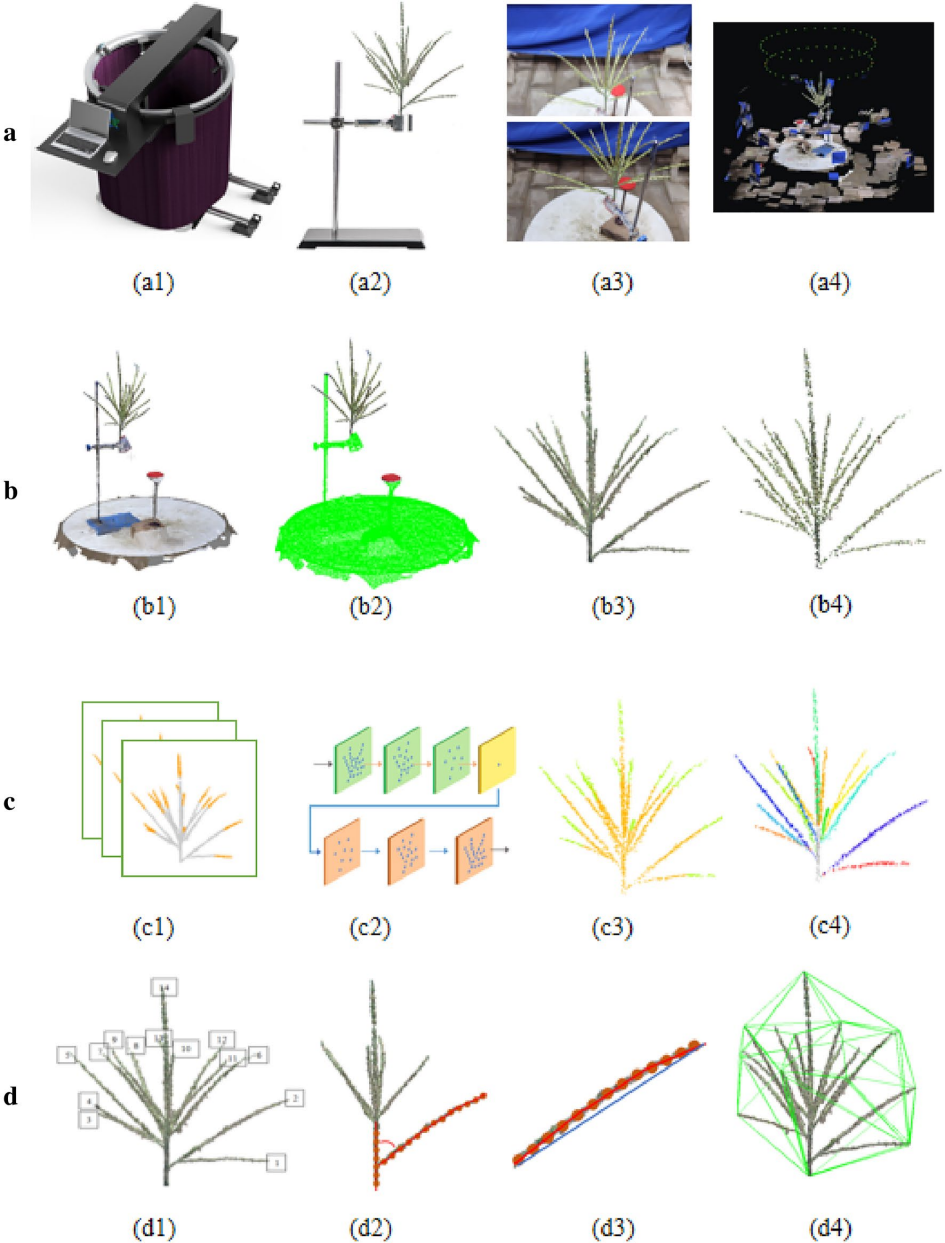
Overview of the proposed methodology. **a** High-throughput data acquisition of maize tassels using the MVS-Pheno phenotyping platform and Point cloud reconstruction: (a1) the MVS-Pheno phenotyping platform, (a2) the iron frame for fixing maize tassel, (a3) maize tassel Multi-view images, (a4) Multi-view reconstruction; **b** pre-processing of point cloud data: (b1) the dense point cloud, (b2) segmentation of maize tassel point cloud and calibration plate point cloud, (b3) the dense maize tassel point cloud, (b4)the down sampling point cloud; **c** the data set construction and deep learning-based segmentation of maize tassels: (c1) point cloud annotation, (c2) the schematic diagram of point cloud segmentation network model, (c3) the point cloud at the top of the branch is segmented through the network model, (c4) Branch extraction; **d** Phenotypic Extraction: (d1) Number of branches, (d2) Branching angle, (d3) Branch bending degree, (d4) Convex hull volume

This study consists of four parts (Fig. 1): high-throughput data acquisition of maize tassels, pre-processing of point cloud data, dataset construction and deep learning-based segmentation of maize tassels, and extraction of the DUS testing traits.

### Materials

Maize tassel samples (180) were selected from the maize association analysis population for the construction of training and test sets to increase the diversity of sample morphological structure. The materials were planted in the experimental field of the Beijing Academy of Agricultural and Forestry Sciences (39°56′N, 116°16′E). The materials were sown on May 10, 2021, based on the unified density (row spacing; 60 cm and plant spacing; 30 cm).

### Data acquisition

**Fig. 2:**
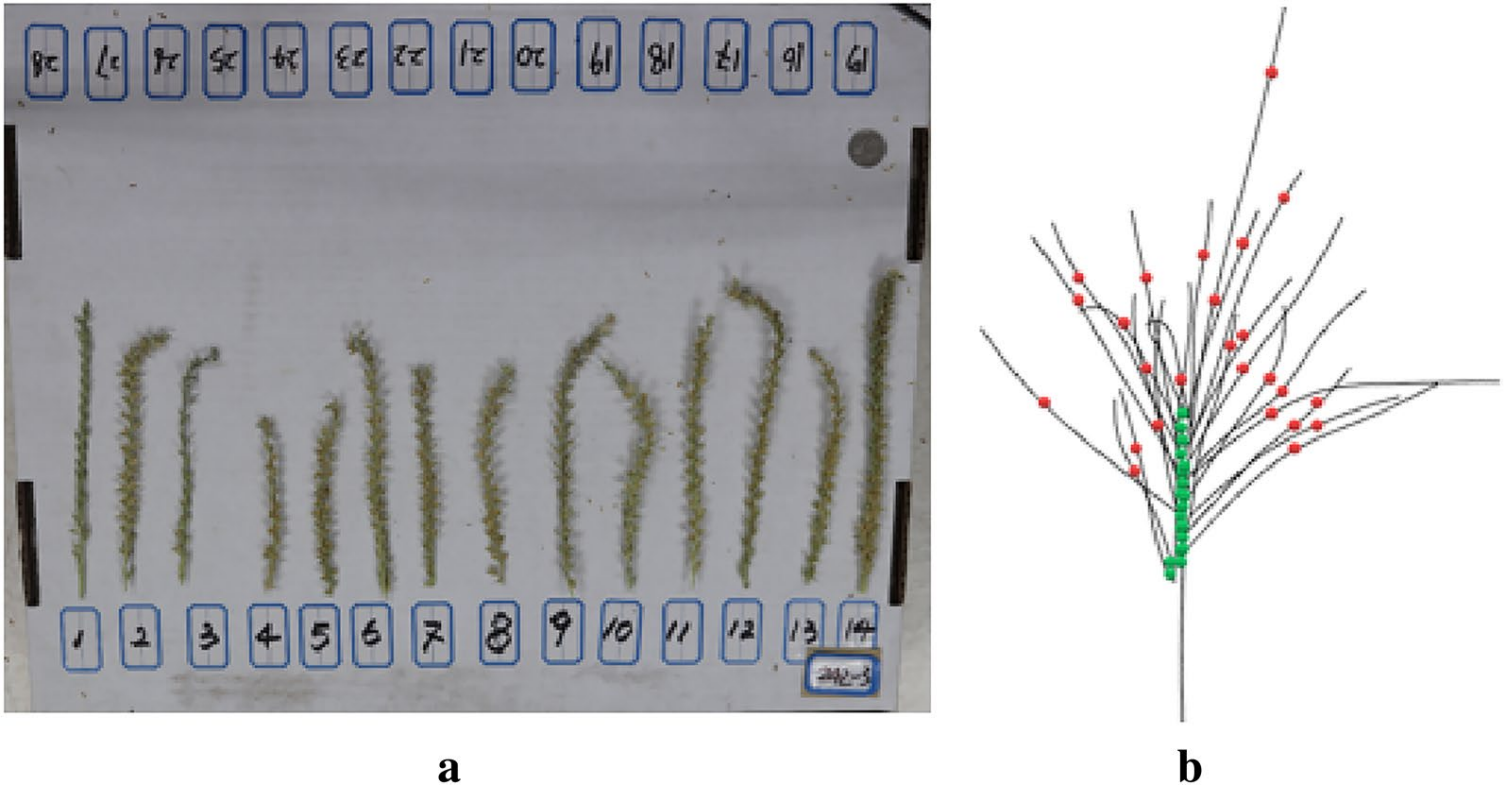
Validation data. **a** Validation data shot. Placement of branches in order of their position on the stalk. **b** maize tassel branch skeleton point, and the red dots represent the upper node, the green dots represent the lower node of the branch

At maize tassel loose pollen stage, the MVS-Pheno V2 platform [35], an easy-to-assemble automated acquisition device with a supplemental light system, was used to obtain multi-view images of maize tassels (Fig. 1a1). The device was deployed in a 3 m long, 3 m wide, and 2 m high tent next to the maize planting area. The maize tassels were cut from the maize plants in the field during the maize power dispersing period, held using a metal frame table (Fig. 1a2), and placed in the central acquisition area of the device for multi-view image acquisition. On the swivel arm of the MVS-Pheno V2 platform, two side cameras(Japan,Canon,Canon77D) are used, which is 80 cm the distance between the cameras and the center of the device,and the vertical distance between the two cameras is 15 cm. For each maize tassel sample, each camera acquires 30 side images at 12° intervals, totaling 60 side images, and each cycle usually takes 90 s (Fig. 1a3). 25 samples of maize tassels ware manually measured to evaluate the reliability of our method, firstly the branch angle and tassel volume ware manually measured by the three-dimensional digitizer device (America, Polhemus, FASTSCAN) (Fig. 2b) [36], and branch length and branch number ware manually measured by image methods (Fig. 2a) [37].

The Structure-from-Motion algorithm (SFM) [38] and the Multi-View Stereo algorithm (MVS) [39] were used for the reconstruction of multi-view point clouds. A batch point cloud reconstruction pipeline system(PC_MVS) was integrated and developed on the basis of the open source libraries openMVS [40] and openMVG [41]. Dense point clouds of corn tassels are reconstructed by PC_MVS (Fig. 1a4).

### Data set production

#### Point cloud pre-processing

The maize tassel point cloud reconstructed using multi-view image data had some noise points. There are three types of noise point clouds, namely, surrounding enclosure and ground noise, support frame and calibration plate noise, and maize tassel attachment noise(color noise and outlier noise). The noise was removed as follows: firstly surrounding enclosure and ground noise point cloud was removed based on the rules of the corn tassel point cloud in the center of the reconstructed point cloud scene (Fig. 1b1); the maize tassel point cloud was separated from the point cloud scene using the HSV space segmentation method of point cloud color information(the point cloud vertex color was converted from RGB space to HSV space, and the threshold masks of H, S and V channels were set) (Fig. 1b2), the threshold values were set as follows: H: 15–180, S:0.05–1, V: 0–1 for each channel of HSV under the conventional indoor lighting environment, and the threshold values could quickly eliminate the shooting background cloth, calibrator point clouds from the scene, and remove the color noise caused by light reflection; the calibration plate point cloud was separated by HLS space, and the maize tassel point cloud is corrected based on the actual size of the calibration plate [35]; the outlier noise points were then removed based on the statistical filtering algorithm as follows:

The point $pi$ was selected from the point cloud $P$, and the average distance between its $n$ neighboring points $\{{m}_{1},{ m}_{2}, {m}_{3}, \dots ,{ m}_{n}\}$ for the point $pi \mathrm{was calculated}$ as follows:

1 $${d}_{mean}=\frac{{d}_{1}+{d}_{2}+\dots +{d}_{n}}{n}$$

where $d$ denotes the distance between two points.

The standard deviation $\sigma$ of that point and neighboring points was determined as follows:

2 $$\sigma =\frac{1}{n-1}\sqrt{{\sum }_{k=1}^{n}{\left({d}_{i}-{d}_{mean}\right)}^{2}}$$

**Fig. 3:**
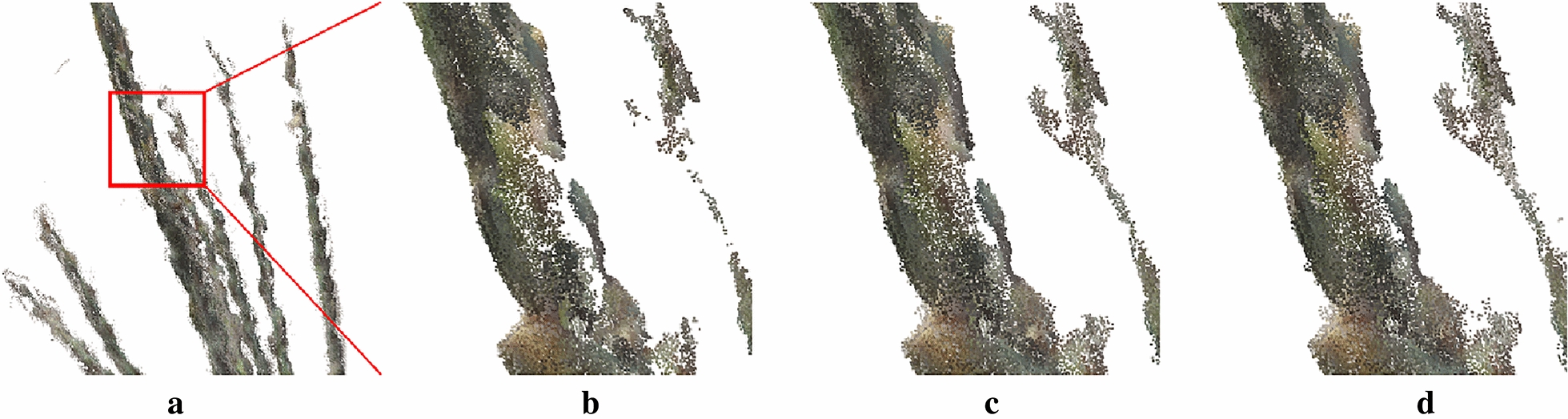
Denoising effect of different parameters. **a** original point cloud; **b** n  = 20, $\alpha$= 0; **c** n = 20, $\alpha$= 0.5; **d** n = 20, $\alpha$ = 1

The neighboring point was removed if the distance from the neighboring point ${m}_{i}$ to ${p}_{i}$ was greater than $\propto$ standard deviations from the average distance $({d}_{i}>{d}_{mean}- \propto \times \sigma$). The noise reduction effect was better when $\propto =0.$ 5 which get a relatively smooth edge while preserving as much information as possible (Fig. 3c).

The point clouds generated via multi-view reconstruction were dense,each maize tassel sample point cloud has more than 500,000 points,which cannot successfully run on the normally configured computer for deep learning training. Firstly,the random sample consensus (RANSAC) algorithm [42] is used, and the sample point cloud has been quickly down-sampled from 500,000 to 100,000 points. Down-sampling using the farthest sampling (FPS) [43] algorithm simplified the number of point clouds without destroying the point cloud distribution, therefore, which was used to further sample the sample point cloud from 100,000 to 40,000 points. In this paper, each sample point cloud is reduced to 4000 points to improve the efficiency of model training (Fig. 1b4).

**Fig. 4:**
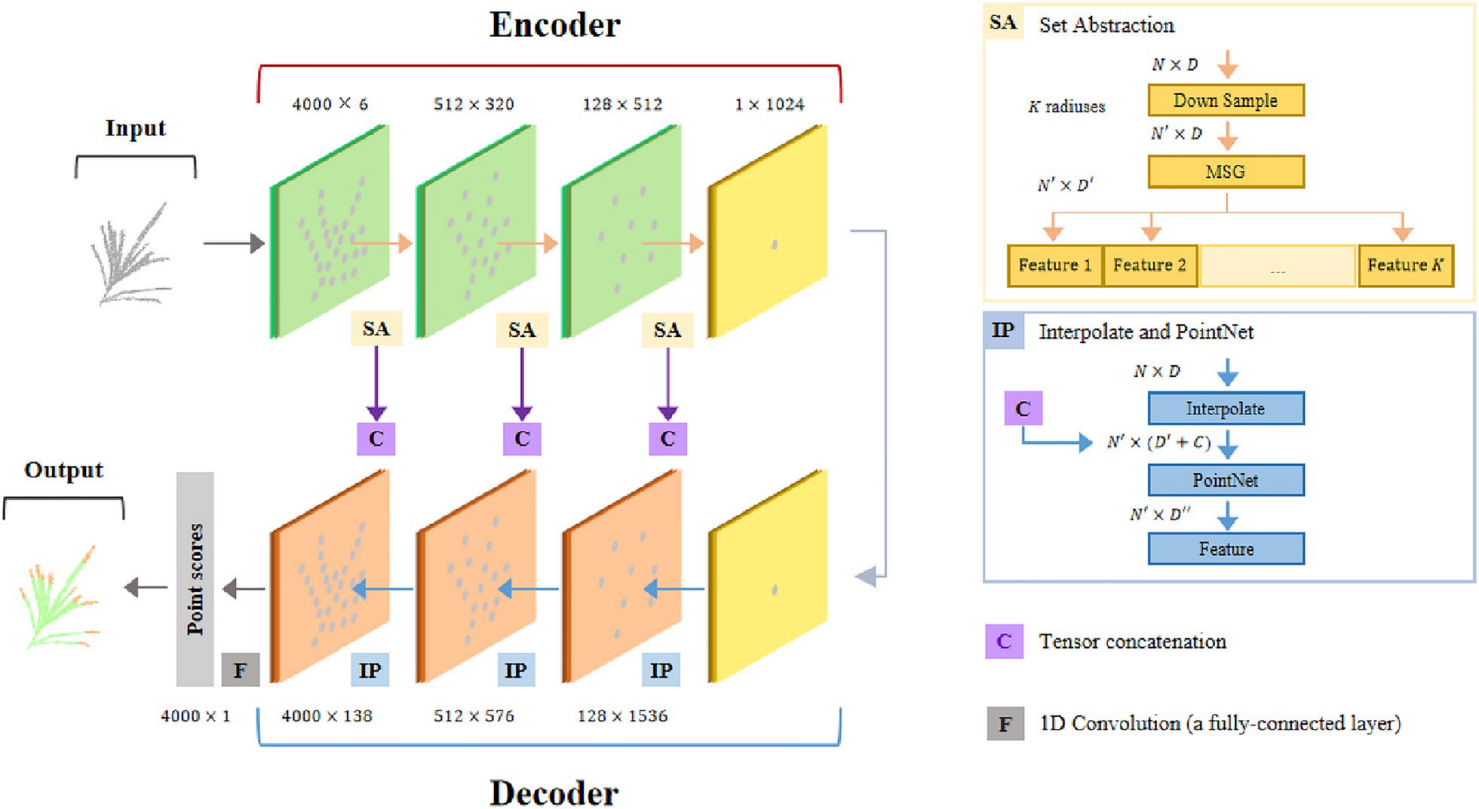
Overview of the network model of maize tassel segmentation. The encoder part consists of three SA layers. Each SA layer sets a different number of sampling points, sampling radius, and Multi-Layer Perceptron (MLP) layer size. The decoder part consists of three IP layers. Each IP layer connects the features extracted by the corresponding SA layer and sets a different MLP layer size

#### Point cloud annotation

Although deep learning algorithms for point clouds have attracted much attention, the methods have not been widely used in plant phenotype processing. This could be because plant point clouds are more complex than buildings, furniture, etc., and there are few open source datasets. Besides, various plants have many differences and thus require more networks to be trained. In addition, manual annotation is a challenge in some self-shading plants. The morphology of maize tassels is divided into compact and spread types. As a result, it is difficult and time-consuming to completely label each branch of the tassel manually. Therefore, only the top point cloud of each branch of the tassel was selected as the point cloud representative of that branch since the roots of tassel branches are compact (Fig. 1c1). The top point cloud of the segmented branch was continuous (3–5 cm long), and did not cross with the main stem of the tassel to accelerate the labeling speed. The branch point cloud of maize tassels was annotated using CloudCompare [44], then the top point cloud of the branch was marked as 1, and the rest points were marked as 0.

#### Dataset enhancement and composition

Data enhancement was performed via the random point sampling method for all labeled branch point cloud data since the maize tassel point cloud data obtained via multi-view reconstruction were dense and non-uniform. In the first stage of downsampling, different initial points were selected to ensure sample dataset in the RANSAC, and different point cloud samples were generated through different initial sampling point sequences. Finally, a new enhanced dataset was obtained, including 1260 training sets, 360 test sets, and 180 verification sets, totaling 1800.

### Maize TasselSeg system

#### Segmentation network framework

The specific network framework used for segmentation is shown in Fig. 4. The network input data contained 3-D coordinate information and normal vector information for N points. An encoder-decoder structure was used to improve the performance of the model for the segmentation task. The encoder module of the network consisted of multiple set abstraction (SA) layers. The point set in each SA layer was adopted and abstracted to produce a smaller scale, larger channel set of points. The SA layer consisted of three parts (sampling layer, grouping layer, and PointNet layer). The sampling layer down samples the point cloud collection using the farthest distance sampling algorithm, and each point sampled is then used as the center of mass of a local domain. The grouping layer constructs a local neighborhood by finding the nearest neighbors around the center of mass sampled by the sampling layer and finally abstracts these local neighborhoods via the PointNet layer. In addition, PointNet +  + proposes two methods, multi-scale grouping, and multi-resolution grouping, which enhance the generalization ability of the network by splicing local features at different scales to better abstract the local features of point clouds. In this paper, the neighborhood features of different radiuses for the center of mass obtained were abstracted using MSG as an extension of SA layer by sampling each sampling layer.

Interpolate and PointNet (IP) layers were designed in the decoder part. PointNet +  + adopts the reverse interpolation method and skip connection to achieve the up sampled point cloud features and obtain the discriminative point-wise feature. Reverse interpolation obtains the interpolated feature with C-dim point feature using the inverse distance-weighted(inverse of distance squared) mean based on k-nearest neighbors (Summation parameter $K$). The feature can be calculated as follows:

3 $${f}^{\left(j\right)}\left(x\right)=\frac{{\sum }_{i=1}^{k}{w}_{i}\left(x\right){f}_{i}^{\left(j\right)}}{{\sum }_{i=1}^{k}{w}_{i}\left(x\right)}, j=1,\dots ,C$$

where:

4 $${w}_{i}\left(x\right)=\frac{1}{{d\left(x,{x}_{i}\right)}^{2}}$$

The local level features were obtained by directly concatenating the representations from the previous encoder corresponding layer onto the interpolated features, which passed through a PointNet to obtain the output. The above process was repeated until the features were relayed to the original point set. Encoder was sequentially performed through the three SA layers to obtain features at three different scales. The decoder was then performed through the three IP layers to splice the features at all scales. Finally, segmentation prediction was performed through a fully connected layer. The model output parameter k for the branch top segmentation of maize tassels was set to 2.

### Loss function

Segmentation networks are essential in the classification predictions of points. Herein, SoftMax cross-entropy function, which is commonly used in classification tasks, was used as the loss function in the training process as follows:

5 $$Loss= -\sum_{i=1}^{n}p\left({x}_{i}\right)logq\left({x}_{i}\right)$$

where $n$, $p\left({x}_{i}\right), \mathrm{and} q({x}_{i})$ represent the number of point clouds input to the network, the probability of the true label of the point, and the predicted value of that point, respectively.

### Network training

The segmentation network was trained using a server with 16 cores and 32 threads CPUs, 128 GB RAM, 1 NVIDIA GeForce RTX 3090 GPU running under Windows 10 operating system, and Pytorch as the training framework.

The point cloud data containing XYZ coordinates, normal vectors, and label values of custom point cloud size were used as the input data for the network training. The training batch size and initial learning rate were set to 24 and 0.001, respectively. The learning rate was reduced by 50% every 20 epochs. ADAM Solver was used to optimize the network. The weight decay of the model and momentum were set to 0.0001 and 0.9, respectively. Network training was terminated when the training loss function was less than the fixed threshold, otherwise, the training continued until all epochs were completed.

### Evaluation indicators

Ground Truth annotation was used to determine the accuracy of the segmentation results based on four quantitative metrics (Intersection Over Union (IoU), segmentation accuracy, precision, and recall). The IoU represents the intersection rate between the predicted and true values of the segmentation network. The accuracy reflects the proportion of correctly segmented points to the ground truth points of the segmentation network. The precision represents the true predicted positive data, while the recall represents the total predicted positive data.

The four indicators were determined as follows:

6 $$IoU=\frac{TP}{TP+FP+FN}$$

7 $$Accuracy=\frac{TP+TN}{TP+TN+FP+FN}$$

8 $$Precision=\frac{TP}{TP+FP}$$

9 $$Recall=\frac{TP}{TP+FN}$$

A point in the maize tassel point cloud was defined as true positive (TP) if it was marked as the same category. A point was defined as a false negative (FN) if it was mislabeled and it was ground truth. The point was defined as false positive (FP) if it was mislabeled and it was not ground truth. Higher IoU, segmentation accuracy, precision, and recall values represented better accuracy.

### Branch extraction of maize tassels

The top point cloud of each branch of the maize tassel was obtained through a segmentation network. Inspired by the shortest path (SP) algorithm [45] and the Median Normalized-Vectors Growth (MNVG)algorithm [46], the bottom-up minimum path algorithm (Algorithm. 1) was constructed, which was used to extract the skeleton point cloud of the tassel and the organs based on the skeleton point cloud for complete extraction of a branch point cloud.
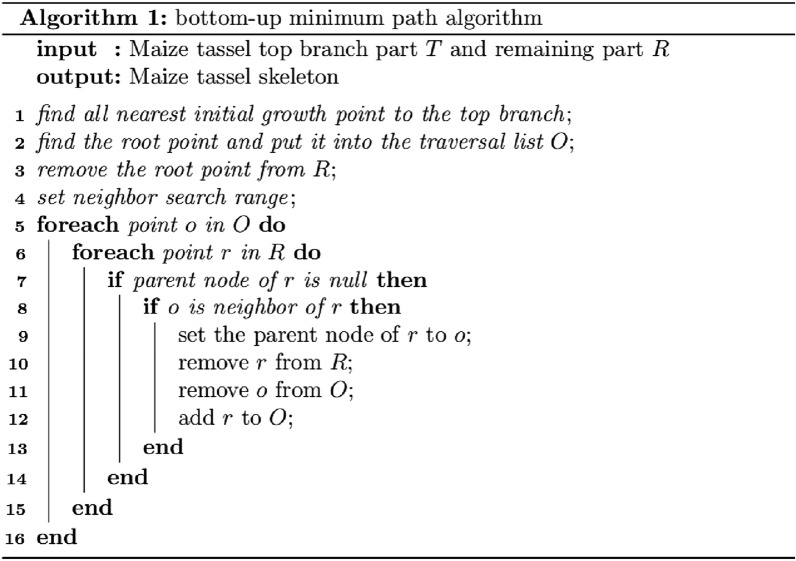


The top point cloud of each branch was obtained by segmenting the maize tassel point cloud using the learned network. The segmented top point cloud was clustered using a density clustering algorithm. The point cloud was considered to have a uniform density since the maize tassel point cloud was down-sampled using the farthest point sampling.

The average distance ${d}_{mean}$ of the point cloud of single maize tassel was calculated, and $\varepsilon =3*{d}_{mean}$ was used as the search radius of density clustering, while $minPts=5$ was set as the minimum density of the neighborhood.

In addition, a threshold of minimum clustering points was selected to reject over-clustered point clouds. The point clouds at the top of the maize tassel branches segmented by the PointNet +  + network were density-clustered to obtain the point cloud instances and the number of branches at the top of each branch.

**Fig. 5:**
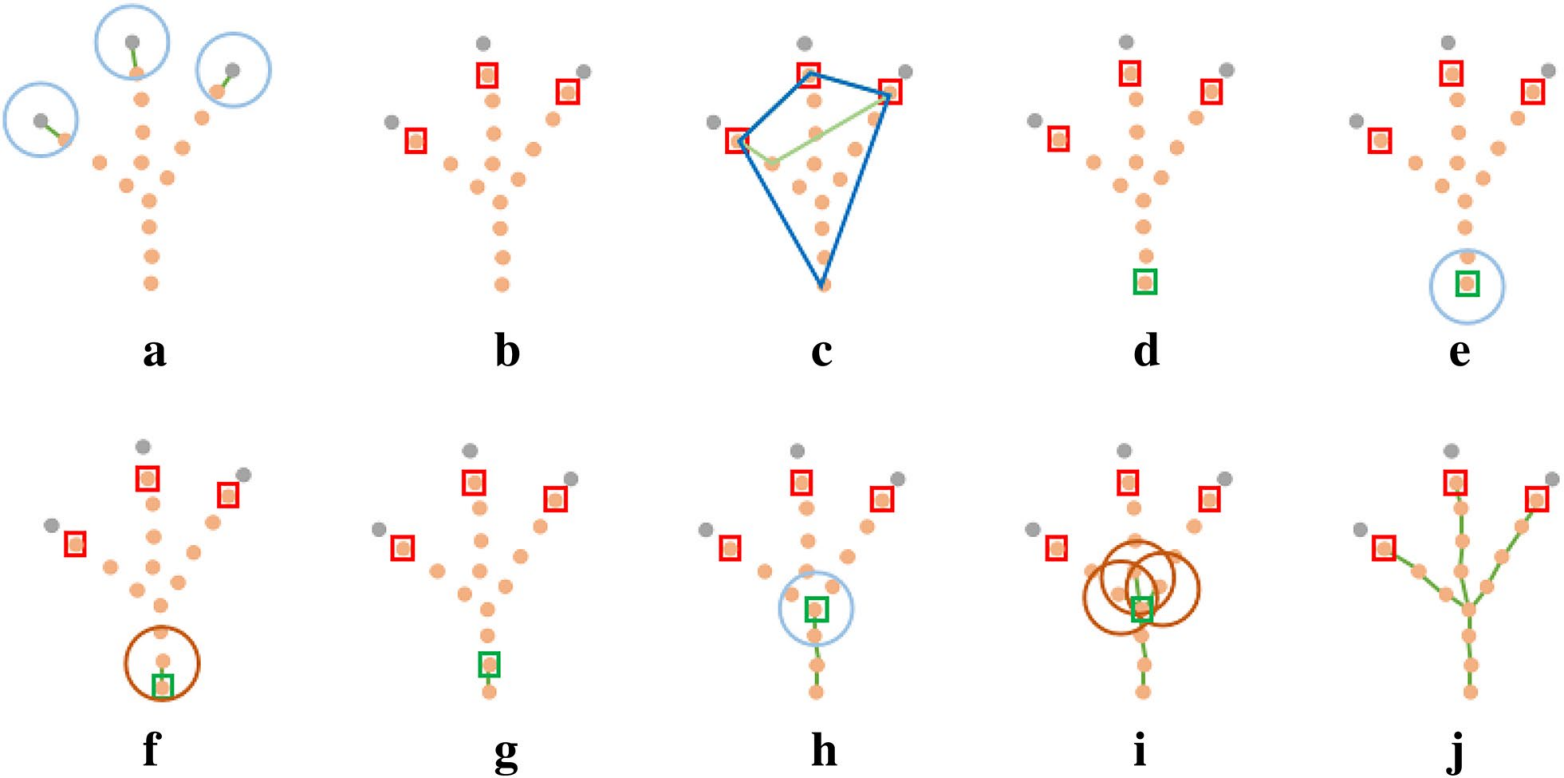
Schematic of the bottom-up minimal path algorithm for maize tassels branch nodes searching. **a** the point cloud is clustered from the network segmentation at the top of branch; **b** the initial growth point at the top of the branch; **c** the nodal convex hull; **d** the root node is selected through traversing the maximum convex hull; **e** the root node is added to the path search queue; **f** the *R* nearest contraction region; **g** the next search node; **h** the fork node; **i** the multiple branch nodes; **j** the multiple shortest path from the branch top nodes to the root node

The bottom-up minimum path algorithm was used to obtain the skeleton of the tassel point clouds, as follows:

First, the maize tassel point cloud was cut into two parts: the top of the branch $T$ and the remaining part $R$. For each branch top instance, the point $p$ in $R$ that is closest to $T$ was searched and saved as the initial growth point at the top of the branch (Fig. 5a, b).

All points $n$ in $R \mathrm{were traversed}$. The volume of the minimum convex hull of $n$ with the initial growth point $p$ of all branches was then calculated. The point $n$ corresponding to the minimum convex hull with the largest volume was selected as the root node of the tassel point cloud. The parent node of the root node was selected as the termination node. The root node was then put into the traversal list $O$ (Fig. 5c–d). The traversal list $O$ was traversed cyclically. Each traversal was performed on all points $p{\prime}$ within the neighborhood $r$ of the current traversal point. The nearest point $p{\prime}{\prime}$ whose parent node was not empty was searched, and its own parent node was set to $p{\prime}{\prime}$. The point $p{\prime}$ was stored in the traversal list $O$, and the current traversal point was removed (Fig. 5e–g). When the traversed point is the fork node (Fig. 5h), multiple branch nodes are searched, and these branching points are set as the parent node respectively (Fig. 5i). This process was repeated until the traversal list $O$ was empty and the parent node information of each point was obtained. Finally, the initial growing point $p$ of the top instance of each branch was searched for the shortest path to get the skeleton of each branch (Fig. 5j). The longest branch skeleton was set as the main stem skeleton. The skeleton points overlapping with the main stem skeleton were set as the stem part. Each original point cloud was judged after obtaining the skeleton point cloud, and its nearest branch was used for fusion. Finally, all branch point clouds were obtained.

The algorithm set a multi-layer traversal neighborhood range to improve the robustness of unevenly distributed tassel point clouds. The initial point cloud was down-sampled to the farthest distance to improve the operation efficiency of the algorithm.

### Extraction of phenotypic traits

Six phenotypic traits, including the number of branches, branch length, branch curvature degree, branch angle, tassel volume, and tassel dispersion, were extracted based on the stem and branch point clouds. The extraction methods are described in Table 1.

**Table 1:** Phenotypic traits calculation method

Traits name	Calculation method	Fig.
Branch count (BC)	Number of point cloud clusters after clustering the results of the segmentation network	Figure 1 d1
Branch length (BL)	Calculate the path length between the two endpoints of the branch	Figure 1 d2
Branch angle (BA)	Angle of branching to main stem formation	Figure 1 d2
Branch curvature (BC)	Ratio of branch length to the distance between the two endpoints of the branch	Figure 1 d3
Tassel volume (TV)	The smallest convex hull of the tassel	Figure 1 d4
Tassel dispersion (TD)	Ratio of the mean value of the tassel branch angle to $\Pi /2$	/

## Results

### Part segmentation results

The segmentation network was trained using 251 EPOCHs and 4000 point clouds. The segmented corn tassel point clouds were quantitatively evaluated. The accuracy of the network rapidly increased in the first 100 epochs, while the loss value rapidly decreased. However, the accuracy and loss stabilized in the subsequent epochs. The highest accuracy and average loss obtained during the training process were 97.69% and 6.30%, respectively, indicating that the model had a good learning efficiency. The final accuracy, recall, and IOU of the completed training model (Table 2) of the top of the tassel branch were 96.29%, 96.36%, and 93.01%, respectively. The segmentation precision, recall, and IOU of the rest were 99.01%, 99.05%, and 94.56, respectively, indicating that the model performed satisfactorily for part segmentation.

**Table 2:** Precision, recall, and IoU of the trained model

	Branch top	Remainder
Precision (%)	96.29	99.01
Recall (%)	96.36	99.05
IoU (%)	93.01	94.56

**Fig. 6:**
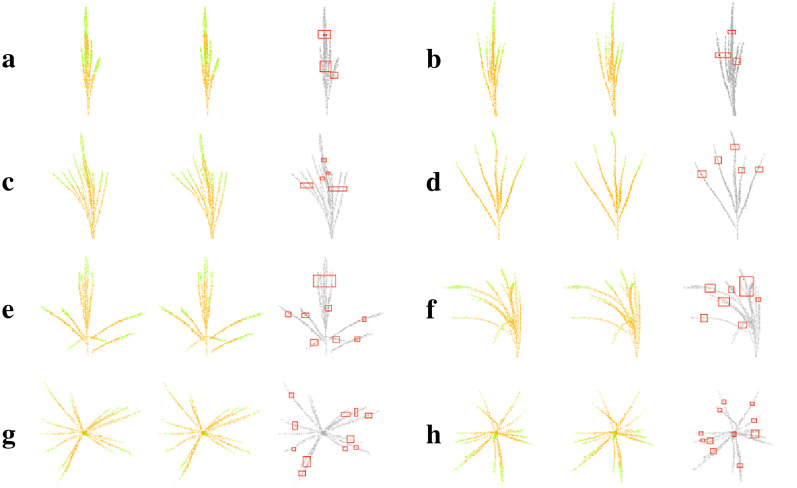
Segmentation results of the test set of maize tassels with different morphologies. Each plot from left to right represents the true label, segmentation result, and error point cloud

The visualization results of the test set segmentation of maize tassels with different morphologies are shown in Fig. 6. Where in, (Fig. 6a–f) show side views of the results of different morphological tassels segmentation, and (Fig. 6 g, h) show top views of the results of scattered tassels segmentation. The segmentation results showed that the model could sufficiently segment the top point cloud of the tassel branch with a few errors compared with manual segmentation. These errors appeared at the boundary between the segmented point cloud of the top of the tassel and the remaining part of the point cloud. The influence of whether the point cloud was correctly labeled at this point on the clustering of the top of the tassel branch was ignored to improve the robustness of the constructed MaizeTasselSeg system.

**Fig. 7:**
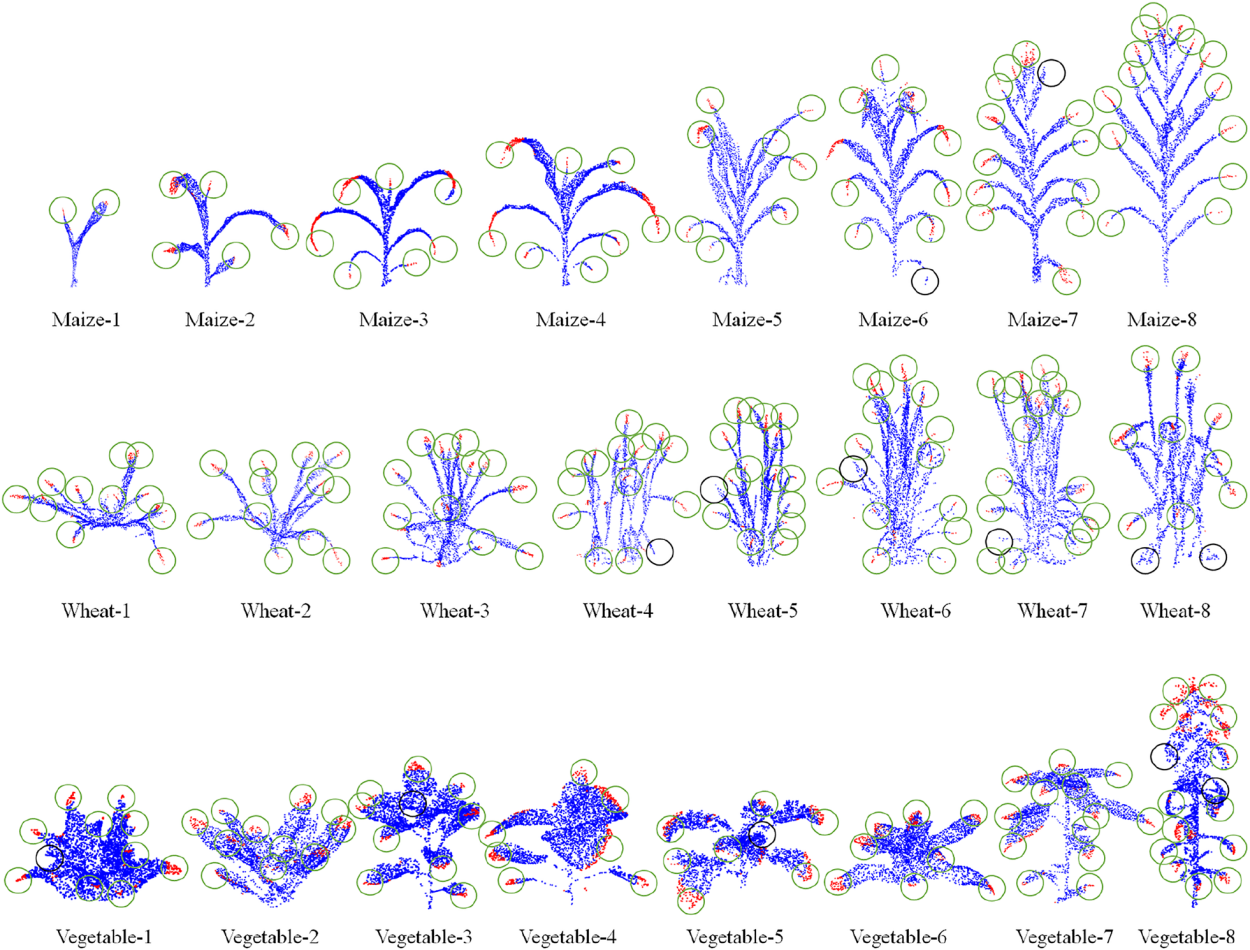
The segmentation results of leaf tip point clouds for three types of plants, namely maize, wheat, and vegetables based on MaizeTasselSeg. Among them, the green circle represents the correctly identified leaf tip, while the black circle represents the unrecognized leaf tip

In addition to maize tassels, there are many types of plants with tip features, such as the leaves of maize and wheat. If the tip point cloud of plant organs can be automatically segmented, then automated segmentation of organ point clouds will become easier. Therefore, more plant with tip features were used to verify the generalization ability of our method. As shown in Fig. 7, the leaf tip point clouds of maize (Fig. 7 Maize-1-8) and wheat (Fig. 7 Wheat-1-8) at different growth stages (from the seedling stage to the mature stage) are automatically segmented, and the leaf tip point clouds of different types of vegetables are automatically segmented (Fig. 7 Vegetable-1-8). Among them, maize and wheat represent fine and long leaves, while vegetables represent large and round leaves. The tip segmentation results indicate that the proposed method has good generalization ability and is suitable for organs point cloud segmentation of various types plant.

### Tassel branch organ extraction

**Fig. 8:**
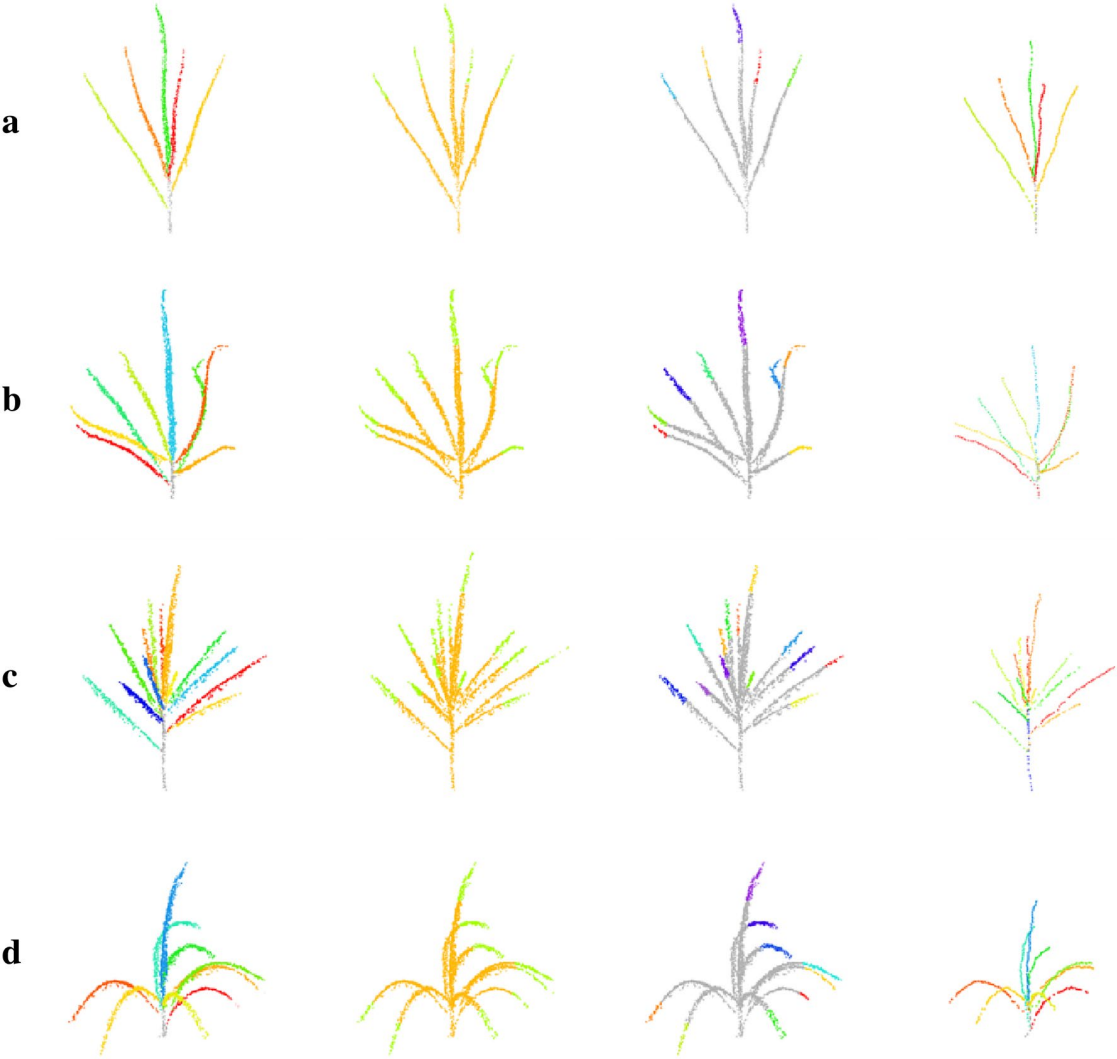
The organ segmentation results of maize tassel point cloud. Images from left to right in each panel represent organ segmentation, network segmentation, branch top clustering, and shortest path extraction. **a** simple and decentralized tassel, **b** tassel bending at the top of the branch, **c** more branched tassel, **d** tassel branching pendulous

The results of branching organ extraction for different morphological maize tassels are shown in Fig. 8. Each row from left to right represents branch segmentation of tassel, part segmentation, top clustering of branches, and shortest path extraction of branches. Visualization results showed that the developed method could adequately segment the branching organs for different morphological maize tassels.

### Accuracy of phenotypic traits

**Fig. 9:**
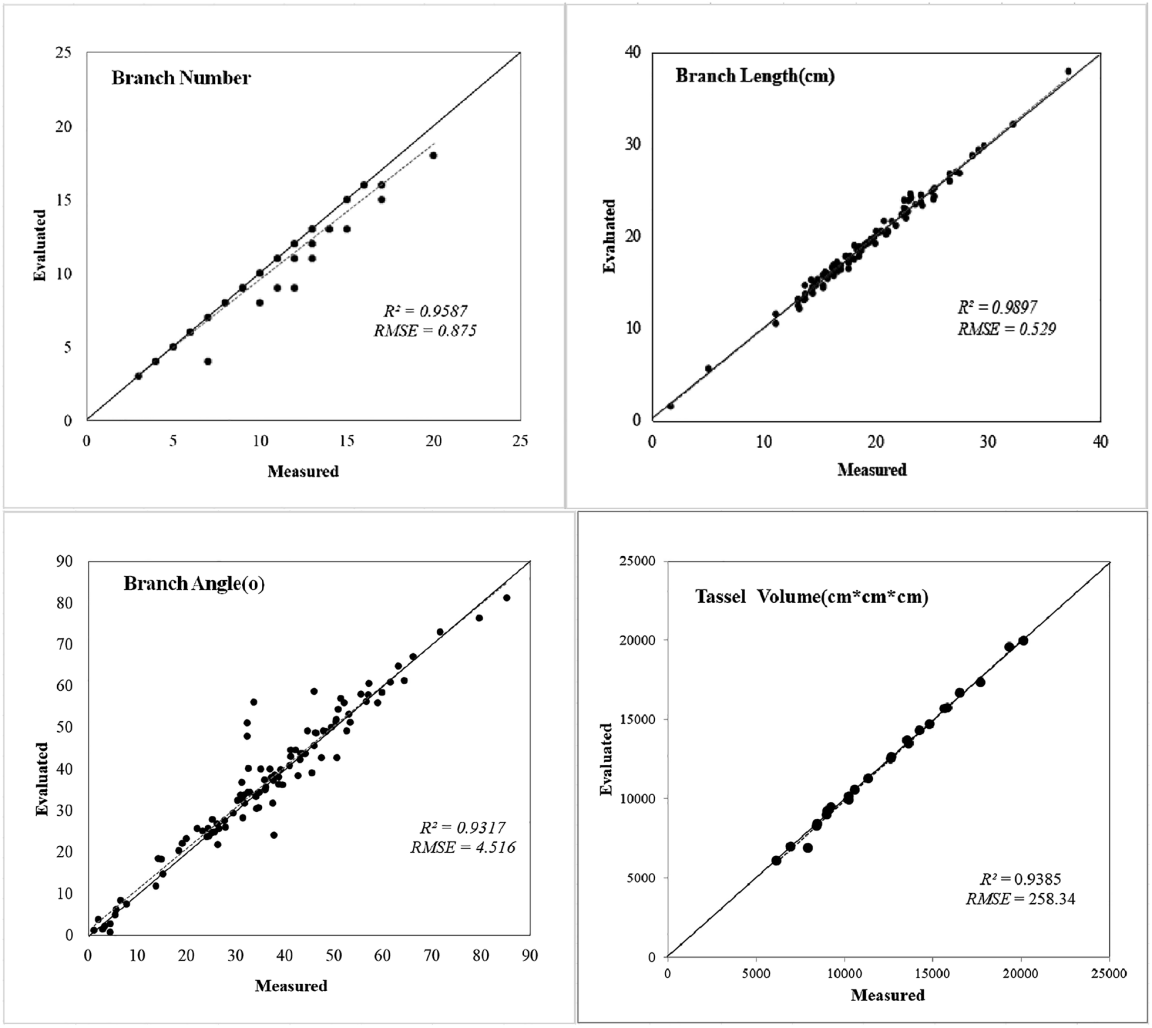
Evaluation of maize tassel phenotypic traits error

Three phenotypic traits (branch number, branch length, branch angle, and tassel volume) ware selected for comparison with the manually measured results. The actual value of the number of branches and branch length ware manually measured by image methods, and the actual value of branch angle and tassel volume ware manually measured through the three-dimensional digitizer skeleton data using CloudCompare. The validation results for each phenotypic traits are shown in Fig. 9. The R^2^ and RMSE were 0.9897 and 0.529 cm, respectively, for branch length, 0.9317 and 4.516°, respectively, for branch angle, and 0.9587 and 0.875, respectively, for branch number, and 0.9385 and 258.34cm^3^, respectively, for tassel volume.

### Varietal analysis of maize tassel phenotypes

**Fig.10:**
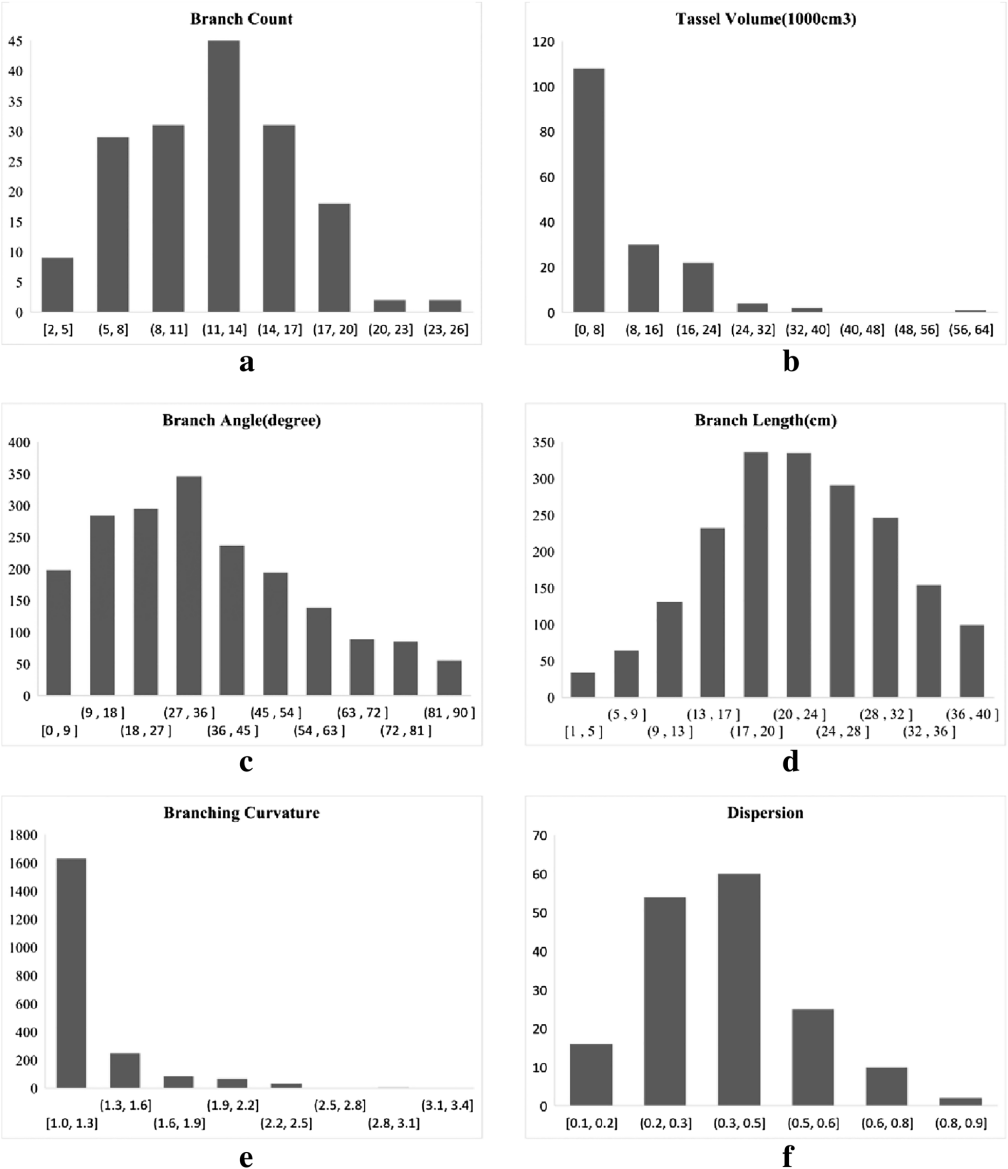
Analysis and statistical results of phenotypic traits of 180 experimental materials. **a** branch count statistics, **b** tassel volume statistics, **c** branch angle statistics, **d** branch lengh statistics, **e** branch curvature statics, **f** dispersion statistics

Organ extraction and phenotypic analysis for the multi-view reconstructed maize tassel point cloud were conducted using MaizeTasselSeg system. The statistical results of phenotypic traits extracted from 180 tassel point cloud data are shown in Fig. 10. Where the units of (Fig. 10a and d) are in cm, the units of (Fig. 10b) are in cubic centimeters, and the units of (Fig. 10c) are in degrees. The horizontal coordinates of all statistics are the corresponding interval values and the vertical coordinates are the corresponding number of branches or the number of tassels. The branch count, branch length, and branch angle were mainly concentrated around 6–16, 20 cm, and 40°, respectively. The branch curvature, tassel dispersion, and tassel volume were mainly below 1.4 level, below 0.5, and below 12000 cm^3^, respectively. The statistical results were consistent with the morphological characteristics of the experimental materials. Therefore, this study provides a reliable and efficient analysis system for the phenotypic analysis of maize tassel.

## Discussion

### Selection of dataset annotation

**Fig.11:**
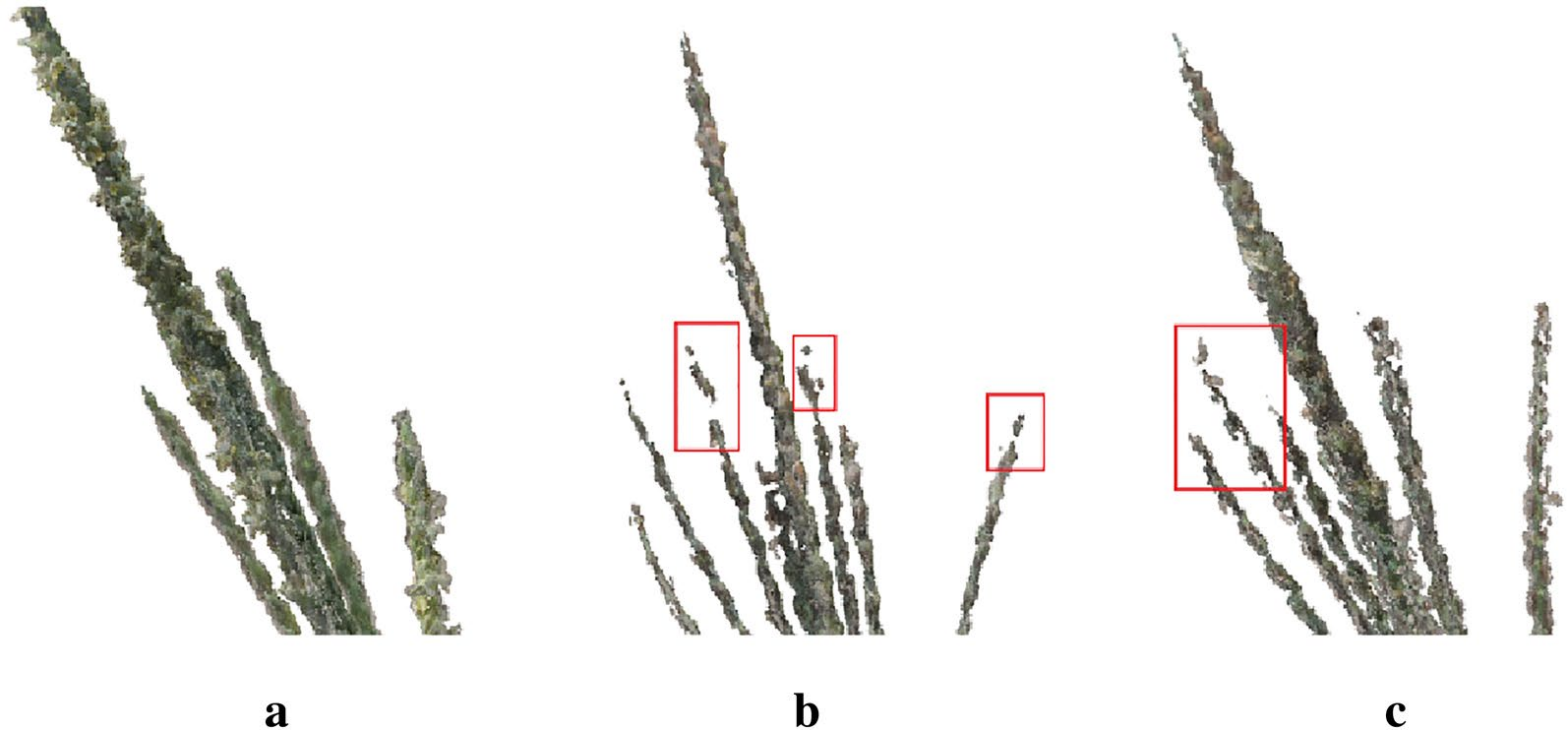
Raw point cloud data quality. **a** no point cloud missing, **b** relatively obvious point cloud missing, and **c** some point cloud missing at the seed-branch junction

The lack of readily available manually-annotated datasets and insufficient software for annotating plant point clouds to reduce labor costs have slowed the progress of deep learning in the plant domain. Maize tassels have thin branches, which are usually tight at the base, which limits the manual production of a complete dataset to segment the branch junctions. The point clouds for compact maize tassels reconstructed from multiple views are often incomplete at these junctions, making it difficult to annotate this type of maize tassel. In this paper, a top-down incomplete labeling method was proposed. The top part of the tassel branch, which is relatively well labeled, was selected for labeling because of the scattered growth structure of maize tassels. A network model was then trained to segment the point cloud of this part. The tassel branch organs were extracted via subsequent clustering and shortest path growth algorithms. However, it is difficult to choose an appropriate annotation scale. Besides, different quality of data has some influence on the choice of annotation scale. Herein, multi-view reconstruction point clouds were the original point cloud data used. Although it has the advantage of the lower acquisition cost, the corresponding point cloud quality is not as good as that obtained via LiDAR scanning. Furthermore, some of the original point cloud data may have discrete missing cases at the top of the branches (Fig. 11). Therefore, it may lead to the wrong learning of the model if only a few point clouds are labeled at the top of the male branch. Finally, the approach of labeling as many points as possible at the top of the male branch was adopted. The part of the labeled points was made in such a way to ensure a certain rigidity (no bending).

### Point cloud sample size

**Fig. 12:**
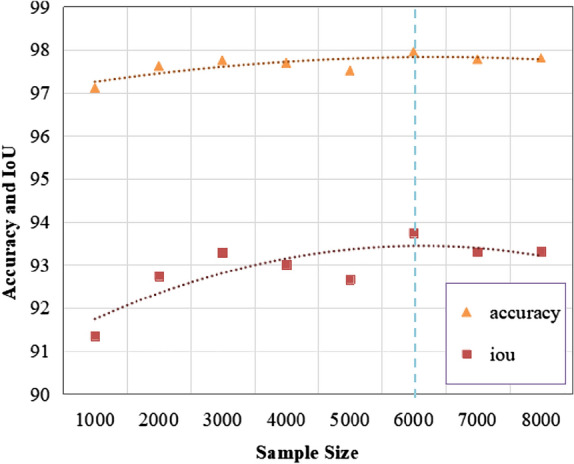
Model training results for different sample sizes

The size of each tassel point cloud in the labeled dataset was 100000 points. The built network model first performed the farthest distance sampling on the input point cloud data. The model parameters were kept constant for different sampling size datasets to analyze the impact of sampling points on the segmentation accuracy of the model (Fig. 12) (sampling size; 1000–8000 and step size; 1000). The accuracy of the model for different sampling size datasets was stable between 97 and 98%. The model obtained the highest accuracy of 97.95% for a sample size of 6000. However, the IoU significantly fluctuated. The model also obtained the highest IoU for a sample size of 6000 (93.75%).

**Fig.13:**
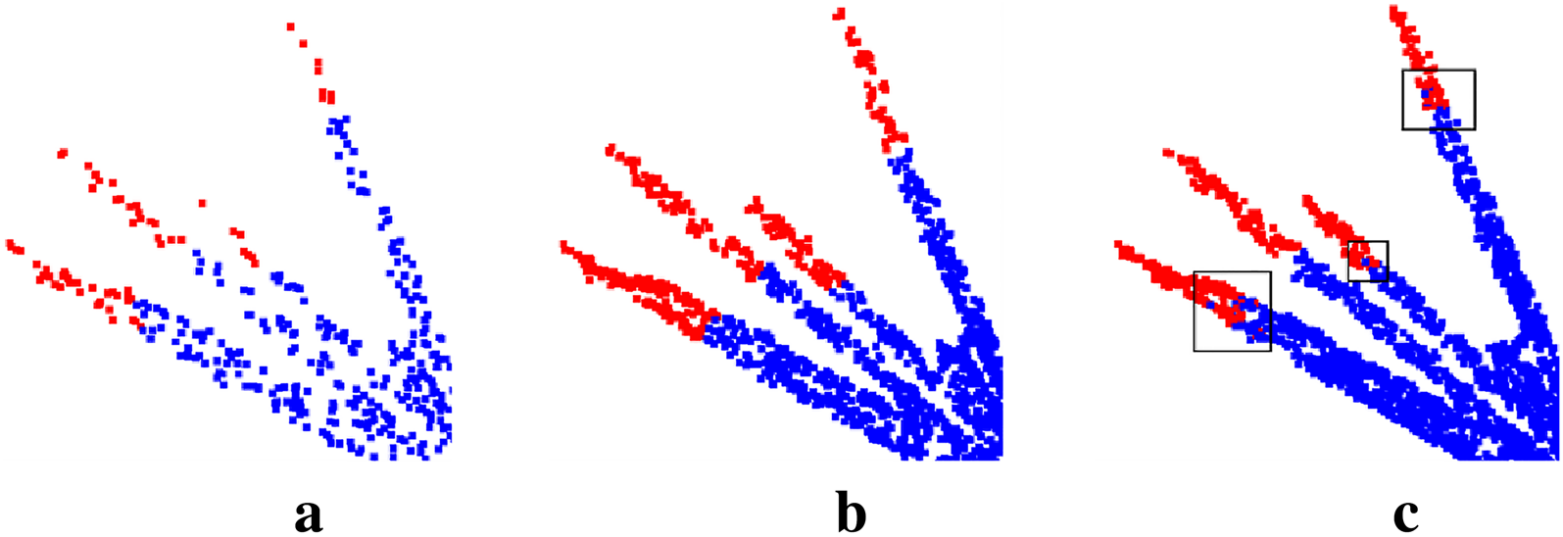
Model segmentation results for different sample sizes. **a** 1000 points segmentation result, **b** 4000 points segmentation result, **c** 6000 points segmentation result

Although the best accuracy and IoU were achieved with a sampling size of 6000, this is not always the case. The segmentation results of the model with sampling sizes of 1000, 4000 and 6000, are shown in Fig. 13. The segmentation results of the top point cloud of the tassel branches at the sampling sizes of 1000 and 4000 did not show a mixture of the two types of markers, indicating that marker error occurred at the boundary between the two marker clouds. However, the segmentation results of the sampling sizes of 6000 showed a mixture of the two types of markers in some of the top point clouds of the tassel branches, possibly due to the uneven density of the original point cloud data that leads to model learning errors. The sampling size also determines the density of the output point cloud. Therefore, the density should be as large as possible to facilitate organ-scale segmentation. As a result, a sampling size of 4000 was selected.

### Problems with compact tassels

**Fig.14:**
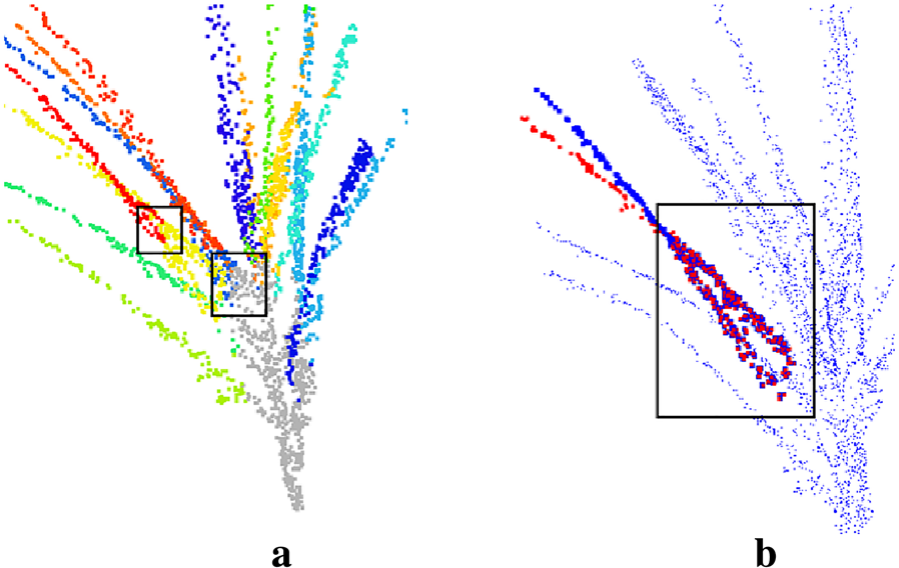
Intersection of compact male spike branches. **a** display of branches by different colors, **b** point cloud of cross-branching common parts

Based on the method of the DBSCAN(density-based spatial clustering of applications with noise) [22], the tassel point cloud branches can be well segmented for maize tassels of dispersed and nearly vertical principal axis, but for compact maize tassels, the effect is poor.Our method is not affected by the growth direction of maize tassels, and is applicable to with compact samples.However, for overly compact samples, the phenotypic accuracy obtained will be reduced. As shown in Fig. 14a, the red and yellow branches cross, causing the shortest paths to merge at the intersection point. The two intersecting branches will share some of the points after organ division (Fig. 14b), where the two intersecting branches share the remaining points after the intersection point. For this type of branching, the branch length, branch curvature, and branch angle of the extracted phenotypic traits will show some deviations.

In addition, the segmentation network was not tested on fully-fitted male spikes, one reason being that the tops of the branches of this type of male spike were difficult to segment manually, so only those spikes that could be manually labeled quickly were experimented with in this paper.

## Conclusion

In this paper, MaizeTasselSeg system was used to process the 3D data based on the 3D point cloud data of maize tassels. The entire process includes high-throughput data acquisition, data pre-processing, data annotation, data set production, learning network model, and organ extraction. Phenotypic traits of interest, including branch number, branch length, and pinch angle, were also obtained. The experimental results showed that the system could accurately acquire phenotypic traits of maize tassels. The system is also fully automated, thus enhancing high-throughput phenotypic analysis of maize tassels. Nevertheless, further algorithms should be developed in future to solve the branch crossing problem in the branches of compact maize tassels and quick labeling for fully adherent tassels.

## Data Availability

The image and point cloud datasets of maize tassels collected from the test sites used and/or analyzed in this study are available upon reasonable request to the corresponding authors. All other data generated or analyzed in this study are included in this paper.

## References

[1] Ninomiya S, Baret F, Cheng Z-M (2019). Plant phenomics: emerging transdisciplinary science. Plant Phenom.

[2] Pieruschka R, Schurr U (2019). Plant phenotyping: past present, and future. Plant Phenom.

[3] Zhao C, Zhang Y, Du J, Guo X, Wen W, Gu S (2019). Crop phenomics: current status and perspectives. Front Plant Sci.

[4] Araus JL, Cairns JE (2014). Field high-throughput phenotyping: the new crop breeding frontier. Trends Plant Sci.

[5] Nikolic A, Andjelkovic V, Dodig D, Ignjatovic-Micic D (2011). Quantitative trait loci for yield and morphological traits in maize under drought stress. Genetika-Belgrade.

[6] Brewbaker JL (2015). Diversity and genetics of Tassel branch numbers in maize. Crop Sci.

[7] Xu G, Wang X, Huang C, Xu D, Li D, Tian J (2017). Complex genetic architecture underlies maize tassel domestication. New Phytologist.

[8] Guan JJ, Zhang P, Huang QM, Wang JM, Yang XH, Chen QB, Zhang JH (2020). SNP markers potential applied in DUS testing of maize. Int J Agric Biol.

[9] Lu H, Cao Z, Xiao Y, Zhuang B, Shen C (2017). TasselNet: counting maize tassels in the wild via local counts regression network. Plant Methods.

[10] Karami A, Quijano K, Crawford M (2021). Advancing Tassel detection and counting: annotation and algorithms. Remote Sensing.

[11] Zou H, Lu H, Li Y, Liu L, Cao Z (2020). Maize tassels detection: a benchmark of the state of the art. Plant Methods.

[12] Lin C, Hu F, Peng J, Wang J, Zhai R (2022). Segmentation and stratification methods of field maize terrestrial LiDAR point cloud. Agriculture.

[13] Gage JL, Miller ND, Spalding EP, Kaeppler SM, De Leon N (2017). TIPS: a system for automated image-based phenotyping of maize tassels. Plant Methods.

[14] Rueda-Ayala V, Peña J, Höglind M, Bengochea-Guevara J, Andújar D (2019). Comparing UAV-based technologies and RGB-D reconstruction methods for plant height and biomass monitoring on grass ley. Sensors.

[15] Hamamoto T, Uchiyama H, Shimada A,Taniguchi R-i. RGB-D Images Based 3D Plant Growth Prediction by Sequential Images-to-Images Translation with Plant Priors; proceedings of the International Joint Conference on Computer Vision, Imaging and Computer Graphics, F, 2020: Springer.

[16] Jin S, Sun X, Wu F, Su Y, Li Y, Song S (2021). Lidar sheds new light on plant phenomics for plant breeding and management: Recent advances and future prospects. ISPRS J Photogramm Remote Sensing.

[17] Su Y, Wu F, Ao Z, Jin S, Qin F, Liu B (2019). Evaluating maize phenotype dynamics under drought stress using terrestrial lidar. Plant Methods.

[18] Liu S, Acosta-Gamboa LM, Huang X, Lorence A (2017). Novel low cost 3D surface model reconstruction system for plant phenotyping. J Imaging.

[19] Yang Z, Han Y (2020). A low-cost 3D phenotype measurement method of leafy vegetables using video recordings from smartphones. Sensors.

[20] Sandhu J, Zhu F, Paul P, Gao T, Dhatt BK, Ge Y (2019). PI-Plat: a high-resolution image-based 3D reconstruction method to estimate growth dynamics of rice inflorescence traits. Plant Methods.

[21] Ziamtsov I, Navlakha S (2019). Machine learning approaches to improve three basic plant phenotyping tasks using three-dimensional point clouds. Plant Physiol.

[22] Dong H, Guijun Y, Hao Y, Chunxia Q, Mingjie C, Weiliang W, Qinglin N, Wenpan Y (2018). Three dimensional information extraction from maize tassel based on stereoscopic vision. Trans Chin Soc Agric Eng.

[23] Wang D, Song Z, Miao T, Zhu C, Yang X, Yang T, Zhou Y, Den H, Xu T (2023). DFSP: a fast and automatic distance fieldbased stem-leaf segmentation pipeline for point cloud of maize shoot. Front Plant Sci.

[24] Maturana D,Scherer S. Voxnet: A 3d convolutional neural network for real-time object recognition.proceedings of the 2015 IEEE/RSJ International Conference on Intelligent Robots and Systems (IROS), F, 2015: IEEE.

[25] Wu Z, Song S, Khosla A, Yu F, Zhang L, Tang X, et al. 3d shapenets: A deep representation for volumetric shapes; proceedings of the Proceedings of the IEEE conference on computer vision and pattern recognition, F, 2015.

[26] Wu W, Qi Z,Fuxin L. Pointconv: Deep convolutional networks on 3d point clouds; proceedings of the Proceedings of the IEEE/CVF Conference on Computer Vision and Pattern Recognition, F, 2019.

[27] Li Y, Bu R, Sun M, Wu W, Di X,Chen B. Pointcnn: Convolution on x-transformed points. Advances in neural information processing systems. 2018; 31.

[28] Qi C R, Su H, Mo K,Guibas L J. Pointnet: Deep learning on point sets for 3d classification and segmentation; proceedings of the Proceedings of the IEEE conference on computer vision and pattern recognition, F, 2017.

[29] Qi C R, Yi L, Su H,Guibas L J. Pointnet++: Deep hierarchical feature learning on point sets in a metric space. Adv Neural Inf Processing Syst. 2017; 30.

[30] Ghahremani M, Williams K, Corke FM, Tiddeman B, Liu Y, Doonan JH (2021). Deep segmentation of point clouds of wheat. Front Plant Sci.

[31] Li Y, Wen W, Miao T, Wu S, Yu Z, Wang X (2022). Automatic organ-level point cloud segmentation of maize shoots by integrating high-throughput data acquisition and deep learning. Comput Electron Agric.

[32] Li D, Shi G, Li J, Chen Y, Zhang S, Xiang S (2022). PlantNet: a dual-function point cloud segmentation network for multiple plant species. ISPRS J Photogramm Remote Sensing.

[33] Turgut K, Dutagaci H, Galopin G, Rousseau D (2022). Segmentation of structural parts of rosebush plants with 3D point-based deep learning methods. Plant Methods.

[34] Dutagaci H, Rasti P, Galopin G, Rousseau D (2020). ROSE-X: an annotated data set for evaluation of 3D plant organ segmentation methods. Plant Methods.

[35] Wu S, Wen WL, Gou WB, Lu XJ, Zhang WQ, Zheng CX, Xiang ZW, Chen LP, Guo XY (2022). A miniaturized phenotyping platform for individual plants using multi-view stereo 3D reconstruction. Front Plant Sci.

[36] Wu S, Wang JL, Zhao YX, Wen WL, Zhang Y, Lu XJ, Wang CY, Liu K, Chen B, Guo XY, Zhao CJ (2022). Characterization and genetic dissection of maize ear leaf midrib acquired by 3D digital technology. Front Plant Sci.

[37] Du JJ, Li B, Lu XJ, Yang XZ, Guo XY, Zhao CJ (2022). Quantitative phenotyping and evaluation for lettuce leaves of multiple semantic components. Plant Methods.

[38] Westoby MJ, Brasington J, Glasser NF, Hambrey MJ, Reynolds JM (2012). ‘Structure-from-motion’photogrammetry: a low-cost, effective tool for geoscience applications. Geomorphology.

[39] Goesele M, Snavely N, Curless B, Hoppe H,Seitz S M. Multi-View Stereo for Community Photo Collections. 2007 IEEE 11th International Conference on Computer Vision. 2007; 1–8.

[40] OpenMVS: multi-view stereo reconstruction library. https://github.com/cdcseacave/openMVS2020. Accessed 20 May 2022.

[41] Moulon P, Monasse P, Perrot R, Marlet R (2017). Openmvg: open multiple view geometry; proceedings of the international workshop on reproducible research in pattern recognition, F, 2016.

[42] Wu Q, Liu J, Gao C, Wang B, Shen G, Li Z (2022). Improved RANSAC point cloud spherical target detection and parameter estimation method based on principal curvature constraint. Sensors.

[43] Kamousi P, Lazard S, Maheshwari A, Wuhrer S (2016). Analysis of farthest point sampling for approximating geodesics in a graph. Comput Geom.

[44] Cloud Compare. https://www.cloudcompare.org. Accessed 20 May 2022.

[45] Vicari MB, Disney M, Wilkes P, Burt A, Calders K, Woodgate W (2019). Leaf and wood classification framework for terrestrial LiDAR point clouds. Methods Ecol Evolut.

[46] Jin SC, Su YJ, Wu FF, Pang SX, Gao S, Hu TY, Liu J, Guo QH (2019). Stem-leaf segmentation and phenotypic trait extraction of individual maize using terrestrial LiDAR data. IEEE Trans Geosci Remote Sensing.

